# The Prophylaxis of Maxillary Development

**Published:** 1912-05-15

**Authors:** N. S. Hoff


### THE PROPHYLAXIS OF MAXILLARY DEVELOPMENT.

BY N. S. HOFF, D.D.S.

The suggestion scarcely needs to be made that any abnormal development or degenerative tendency in the typical maxillary bones and the teeth will menace the normal health and furnish predisposing causes of disease in the dental and oral tissues. In fact among the earliest attributed causes of decay of the teeth, and which are still quoted in every classified statement of dental diseases, may be found failure of perfect calcification and abnormal articular relations. In recent years we are suggesting dental diseases as probable causes of many other affections located in their contiguous regions. Disease of the eye, ear, lungs, intestines, &c., may have their indirect if not direct source of infection in the mouth. For our present purpose we shall only call attention to the main causes producing mal-occlusion of the teeth, that we may present such prophylactic suggestions as are applicable in preventive treatment.

The most careful observers and writers on mal-occlusion of the teeth say that typically normal occlusion is rarely seen and that we are well content to approve of the practically normal and to use it as a working if not an ideal standard, especially in the permanent dentition. After closer study and experience we are now convinced that the deciduous dentition, which we have heretofore considered generally normal, is only normal in about 60 per cent. of the cases which come under the observation of the Orthodontist. It is of course more difficult to establish a normal type in the deciduous than in the permanent denture. We shall however, largely confine our considerations to the permanent denture, except where this may be influenced by some condition of the deciduous denture.

In view of the statement made above that the normally typically is so rarely seen, we may ask how it is possible to know the normal, and why it is that more of the normal does not exist? We can answer this in a few words by saying that there are two great causes of mal-position of the teeth in the maxillary arches. One of these is to be found in arrested development of the bones of the jaws and face, and the other in a variety of events associated with the growth of the teeth and jaws which may be generally classified as accidental. These two causes are so generally present in our modern civilization that to-day as never before we are studying the problems of right living with more scientific care than ever before in the history of the world. Modern physicians and dentists have caught the hint and are fast turning their attentions to the problems of sanitation and preventive treatment. We may not expect a radical change and there will always be a place for the healer and surgeon, as some will be born wrong, and it is much easier to live in luxury and ease without thought as to right conduct even though we know the unchanging character of natural law. But we are a progressive people, and some of us are wise and heroic enough to live the truth when we know it, or are forced to do so. The ideal dentistry of the future will be preventive.

As we can best present our subject by practical illustration we shall use the logical material bearing directly on the subject. The first molar is the first tooth of the permanent denture to develop and erupt into the dental arch. The lower molar usually erupts first at about the sixth year. As this tooth erupts back of and free from the diciduous arch and from a portion of the jaw which before had contained no tooth, we suppose that it should always erupt in its normal position, and some authors maintain that it always does this, it probably does with more uniformity than any other tooth. Dr. Angle was so confident of the normal location of this tooth that he made it the basis of his method, or system, of correcting mal-occlusion of the teeth. For these reasons and for technical and scientific purposes this tooth becomes immensely important as it is made the very foundation of the permanent arch. Its preservation in the very best possible condition of integrity and health is demanded by the orthodontist. It may also be noted that functionally this tooth receives the burden of the process of mastication.

PROPHYLAXIS OF THE FIRST MOLAR. This tooth is more often seriously diseased or lost entirely than any other tooth. It erupts so much earlier than any other tooth and so far back in the mouth that many parents are not aware that it is a permanent tooth. Therefore they neglect to have it given proper care until it becomes seriously diseased, so that it can never be made functionally satisfactory, and many times it has to be extracted. Its mal-position is usually so slight that the average dentist will not notice it or call the patient's attention to it. Its more common mal-position is the tipping of the occlusal surface to the lingual and the drifting of the entire tooth lingually. If the deciduous molars had decayed on their proximal surfaces, or been prematurely extracted, it will be found to have settled forward. Slow or delayed development occasionally will occur because of a local or systemic disease which causes interference with the circulation and nutrition and may even stop the process of eruption entirely. Such accidents are liable to cause the tooth to drift into a submerged or torsal mal-position or mal-alignment.

If patients can be put under prophylaxis treatment at an early period practically all such conditions can be avoided as they are wholly preventable. All children should be under prophylaxis surveillance at least at two years of age, and at the age of four or five they should have systematic treatment. The method and importance of prophylaxis treatment for the deciduous denture we have already discussed, its results will be appreciated more fully in connection with the period we are now considering. Systematic prophylaxis should be maintained throughout the whole period of changing from the deciduous to the permanent denture. By this we mean that the teeth should not only be kept clean, but that suitable spaces should be provided for each incoming tooth, even should this necessitate expansion of the arch.

It is not usual that any special attention should be given to preparing the way for the first molar as there is space for its eruption, but it should be carefully inspected and in case there is delay or evident impaction, means for its easy and proper emergence should be adopted. Ordinarily this may be accomplished by a systematic course of massaging the overlying gum tissue with the finger or a vibrator or the application of stimulating lotions applied to the overlying gum to promote absorption. In difficult or specially delayed emergence surgical interference is indicated in the form of lancing or cutting away the tough or fibrous membrane overlying the tooth which is not absorbing normally.

If, as the tooth emerges it is apparent that it will not properly occlude with its antagonist in the opposite jaw, it should be watched carefully and, if necessary, such orthodontic treatment as may seem desirable applied as will help to guide the tooth into its proper alignment. A slight interference at this time will often prevent more extensive orthodontic treatment later. Inclined crown surfaces may be tipped up to the normal position by rubber elastics fastened to the buccal surface of the upper molar and extending to the lingual surface of the lower molar for instance, although in severe cases it will be wiser to place an arch or bands and wire brace combining several teeth for a firmer and more fixed anchorage to work from and avoid changing the anchor tooth. Such simple treatment will establish at the proper time, normal occlusal relations and may prevent torsal or mesio-distal mal-occlusions and firmly fix the teeth in their proper relations for all time. These teeth once properly placed the function of mastication is at once established on a normal basis and the stimulus given to the peridental tissues insures proper development of the alveolus and jaw. No greater pleasure will be realized by these little patients than that which comes to them from their ability to naturally close their teeth together, and especially in the act of masticating their food completely. This is the first and most important step in securing complete normal occlusion and neglect to adjust these teeth in their right relations at this time is almost necessarily fatal to future normal development.

Logically we should perhaps stop here and discuss the repair and preventive treatment of these teeth, but we shall postpone that for the general discussion of the technique of prophylaxis. In order that we may discuss the subject by periods, we shall briefly consider the influence of retarded development of the bones of the face and the jaws as factors in producing mal-occlusion of the teeth.

RETARDED MAXILLARY DEVELOPMENT. Older students of orthodontia seem to have concluded that dwarfed maxillary bones were in large measure due to the fact that the teeth failed to erupt into their normal places and consequently there was no stimulus for complete development. We however know that other powerful agencies are at work in the developmental period that control the development of the bones of the face as well as of the jaws, and we believe also that it is practicable to overcome these forces if the proper preventive treatment can be applied at the right time. The most commom of these disturbing factors in arrested development is Mouth Breathing, from diseased tonsils and adenoid growths in the pharynx and post nasal regions.

MOUTH BREATHING. The detrimental influence of mouth breathing in producing mal-occlusion of the teeth, and especially those cases having very narrow arches and high vaults, seemed to be so obvious that orthodontists have for a long time attributed these defects to this cause. The fact is however that mouth breathing is only the general symptom or manifestation of more obscure and primary affections which must be considered and changed before any satisfactory remedial treatment of permanence can be adopted.

We are accustomed to think that the first year of infant life is the period of greatest stress, because of the large mortality of the period, which is undoubtedly due to the problem of finding a proper artificial dietary for the mothers insufficiency, and also to our deplorable lack of practicable knowledge of infant hygiene. In addition we are accustomed to attribute much of childrens illness at this period to the development and eruption of their first teeth, a process which ought to be so normal that there should be no such stress as to make it a factor in the child's ill health. Next to this stress period in the childs life none is of more concern than the period of second dentition which begins about the time of the eruption of the first molar tooth and lasts to the age of puberty or to about the fourteenth year. During this period not only are the teeth erupting, but the facial and jaw bones with their mucous membranes, the glandular organs of the mouth, nose and throat are also maturing. It is highly desirable that every one of these tissues shall be as normally perfect in form and structure as possible, for in the future they are to have most important functions to perform and will also be subjected to the most trying ordeals. They are to guard the gateway to the vital organs of the entire system, and all the air and food that passes them must be adequately inspected and prepared for assimilation by the highly specialized internal organs. We may therefore believe that any extra care that we may take to put these organs in the best possible form to accomplish their work will add materially to the general health and well being of our patients.

THE TONSILS. The faucial and pharyngeal tonsils are the undoubted cause of mouth breathing and require special attention in all prophylaxis measures. Medical writers have discussed the functions and diseases of the tonsils from time unknown almost, and even to-day we have no universal agreement as to the diseases of these organs and their treatment. Dental writers have long believed the tonsils responsible for many dental diseases, and have advocated their entire removal in early life. The general belief is that they harbor in their crypts infection which readily spreads to the mouth tissues. It is undoubtedly true also that they become diseased in child life, and on account of their related vascular and nervous supply they become primary sources of actual interference with the normal growth and development of the teeth and jaws. It may be cited in illustration, that because the mandible is undersized that its undeveloped condition was directly due to the fact that the deciduous teeth were neglected and lost prematurely, so that the lateral pressure of contact being lost there was no stimulus for bone growth. The probabilities are that the most potent factor in this degenerative event was caused directly by diseased and hypertrophied tonsils which rendered complete closure of the teeth impracticable without pain and discomfort to the patient, which a child would naturally avoid. Such a condition in the faucial tonsils would soon produce the open mouth habit in any child in spite of all effort on the part of the parents to overcome it by any of the usual moral persuasion methods. The entire nutrative process of the mandible might be checked because of the nerve aberation produced by these diseased tonsils, which are kept in this condition by their constant and unnatural exposure to the irritating unfluence of mouth breathing. It may be readily inferred that other tissues besides the teeth are affected also by being placed under the bad influence of an irritable nervous system, and that no normal development takes place except as a result of inherent cell predisposition. Pruning and cultivating our fruit trees does not insure us sound fruit, we are compelled to shield them from such accidents as worms, bacteria, scale and the elements which, with various other menacing conditions afflict them. So when we are growing teeth and jaws we must furnish a proper environment if we are to produce the typically normal. If the oral structures are to be normally developed we must see to it that their contiguous tissues are in normal condition; we should not expect jaws of normal size, or good teeth normally placed in the arches, or gums that are capable of resisting the friction of mastication without irritability, if developed under bad influences. It cannot be expected that teeth developed with such a handicap will be normal in type, size or structure; and almost without exception under these conditions we find abnormal occlusion, sometimes to an alarming extent. The inference is that diseased tonsils are a menace to the normal development of the teeth and jaws, and they should receive radical attention before they shall have caused a serious and impossible recovery. We have seen cases where extensive orthodontic treatment was without desirable results because the tonsils had not been taken into consideration at the beginning of the treatment. If it is so necessary that the tonsils receive preliminary attention for orthodontic treatment how much more should it be done as a prophylactic measure. The equilibrium of influence in the developmental process is so very slight in many cases that the most critical diagnosis and the application of a properly balanced treatment at the proper time is of the utmost importance.

ADENOIDS. Associated with the upper jaw we have the pharyngeal tonsils to contend with, and they exert a nore powerful force on the upper jaws than the faucial tonsils do on the lower jaws in retarding and perverting development. These glands are located on the upper surface of the soft palate at the distal ends of the nasal passages. Because of their adenoid character they are very liable to infection and disease, but they are more generally affected with a kind of vegatoid hypertrophy. To such an extent are they liable to this growth that it frequently fills the post nasal space and practically precludes nasal breathing, or at least with so much effort that the patient prefers mouth breathing. This adenoid growth is always attended with more or less nervous irritation, due in large measure to the constant and conscious effort required to breathe normally. This general nervous irritation has a pronounced reflex effect on the general mentallity whose function is perceptibly affected, so that dulness and even stupidity are frequent manifestations. The development of the bony structures of the face and head suffer probably more than the teeth or other structures, and the bones of the nose are invariably either incompletely developed or distorted and sometimes hypertrophied. The teeth are therefore compelled to develop in most unfavorable surroundings, and to find a suitable space in which to establish themselves is the reason for the orthodontist.

Post-nasal adenoids and enlarged faucial tonsils are almost always associated with mouth breathing, and patients so afflicted can neither eat nor breathe with comfort or profit, and the continued strain soon produces a depressing effect on the whole nervous system. Such patients not only fail to get proper nourishment but may start on a degenerative career that will end fatally. Whereas we believe that growing children should have an excess of food for selection we have in these conditions a practicable impossibility for the patient to get sufficient food for actual sustenance, and the result can only be disastrous. Children suffering from these troubles are peevish and irritable and slow or actually stupid in their school work, and in some cases they become moral and physical degenerates, and it all is not because the child is a mental degenerate but simply because he has not had a chance to develop the organs most necessary for his proper development and physical well being,—and it all could be prevented.

These conditions are always the direct cause of exaggerated dental deformities, such as crowded arches and narrow jaws and high vaults, and the bones of the nose and face are out of harmony and in some cases actual deformities exist. In such cases if left to full development there is small chance of either facial or dental correction. The conditions of the nose and mouth are most favorable for infection which is liable to extend into all the facial sinuses and to the middle ear causing eye and ear troubles of the most aggrevating character.

The teeth practically always develop to their normal size, and however little the degeneracy of the jaws there will be an inevitable crowding which they can not overcome, and a distortion of position which is unnatural and noticable. A single tooth erupted in abnormal position will so throw the occlusion out of adjustment that the function can be had only with a conscious affort on the patient's part, which of course is unfortunate. It is surprising how slight a defection will sometimes disturb the automatic function of mastication. Absolute co-ordination of every tooth and every cusp is more essential to comfort and happiness than we are inclined to believe. It is almost as essential as that the cogs of the watches wheels shall match exactly to keep the machine running regularly and without friction and disastrous consequences. When the teeth do not occlude accurately there necessarily result vicious forces which not only crowd the teeth out of their proper places and relations but also wears their cusps irregularly, to say nothing of the pulp and peridental disturbances that many times follow. Teeth that are badly occluded are a constant irritation, and such patients will not make the use of them as they otherwise would, and as a consequence the natural cleansing of their surfaces by vigorous mastication is lost. Many a patient with a badly adjusted occlusion will stoically endeavor to masticate his food in spite of his inability, and believes that he is succeeding but he can not keep his conscious mind always on the act, he forms an unconscious habit that because it is not natural, is a constant irritant to his nervous system which in time will express itself in some definite abnormal function. The result is a neurosis, with a series of pysical break downs that the puzzled physician can not account for, because he has become accustomed to let the dentist take care of the teeth, and he thinks we have done so. This condition which is practically universal in some degree is preventable, and if medical and dental practitioners would work together and they could have entire control of the children, there can scarcely be any doubt that within a generation of rigid enforcement of oral prophylaxis principles, normal occlusion would be the rule rather than the exception, and the large majority of children's diseases would be exceptional rather than prevalent as at present. If this could be done a race of physically perfect men and women would be given to the world who would be capable of coping with our modern civilization and its strenuous demands.

Medical and dental writers of recent times have advanced the theory that pulmonary tuberculosis was often developed as a direct consequence of oral and nasal mal-formations, not alone because of the greater opportunities for infection, but because all the respiratory accessory organs and tissues are degenerated to an extent which precludes full function. Such under-developed organs are not even capable of their own full functions and naturally they possess no reserves for defense, and in place of guarding the gateway of life they serve as the chief means of introducing into the system fatal infection. If tubercular infection can be introduced into the system by such means why not other pernicious diseases? The time for dental prophylaxis is of course during the developmental period and the earlier the better. The maxillary bones stop developing at about the thirtieth year, but of course they may cease at any time, and so it should be our aim to co-operate with the natural forces early to secure the most effective results. The best results should be had so far as the eruption of the teeth is concerned by beginning not later than the sixth year and keeping it up to the fourteenth or sixteenth year if necessary.

